# DYADIC AND LONGITUDINAL STUDIES OF CLOSE RELATIONSHIPS: NEW INSIGHTS INTO HEALTH AND WELL-BEING ACROSS ADULTHOOD

**DOI:** 10.1093/geroni/igac059.413

**Published:** 2022-12-20

**Authors:** Gloria Luong, Christine Fruhauf

## Abstract

This symposium includes 5 papers using intensive, longitudinal, and/or dyadic data on social relationships, revealing novel insights into health and well-being across adulthood. Fruhauf and colleagues conducted a feasibility and acceptability to test their Merging Yoga and self-management to develop Skills (MY-Skills) intervention, which targets caregiving dyads (caregivers and care receivers) dealing with persistent pain. Participants completed group interventions meeting twice a week for eight weeks, with each two-hour session including self-management education followed by yoga. A priori benchmarks for feasibility were met for the dyads, suggesting that these interventions may lead to improved well-being for caregiving dyads. Turner and Hooker conducted a 30-day microlongitudinal study of middle-aged daughters who are caring for their parents with dementia. On days when caregivers had more positive appraisals of caregiving than their own average, they had greater physical activity goal pursuit, independent of that day’s negative appraisals. Luong and colleagues also leverage an intensive longitudinal study spanning 3.5 month that prospectively tracked older adults and their close partners as they transitioned into senior housing facilities. Well-being varied by marital status, and changes over time varied by age. Weidmann & Chopik examined older romantic couples from 36 nations and found that between-partner correlations between depressive symptoms, self-rated health, and cognitive performance diffed across cultures. Finally, Purol et al. found that among middle-aged and older couples, similarity effects of personality facets on health and well-being were relatively small in magnitude, suggesting personality similarity among couples may be overstated in the literature.

